# A novel exploratory chemometric approach to environmental monitorring by combining block clustering with Partial Least Square (PLS) analysis

**DOI:** 10.1186/1752-153X-7-145

**Published:** 2013-08-30

**Authors:** Dragos V Nica, Despina Maria Bordean, Ioan Pet, Elena Pet, Simion Alda, Iosif Gergen

**Affiliations:** 1 Banat’s University of Agricultural Sciences and Veterinary Medicine from Timisoara, Faculty of Animal Sciences and Biotechnologies, Timisoara, Calea Aradului 119, RO 300645, Romania; 2 Banat’s University of Agricultural Sciences and Veterinary Medicine from Timisoara, Faculty of Food Processing Technology, Timisoara, Calea Aradului 119, RO 300645, Romania; 3 Banat’s University of Agricultural Sciences and Veterinary Medicine from Timisoara, Faculty of Agricol Management, Timisoara, RO 300645, Calea Aradului 119, Romania; 4 Banat’s University of Agricultural Sciences and Veterinary Medicine from Timisoara, Faculty of Agriculture, Timisoara, RO 300645, Calea Aradului 119, Romania

**Keywords:** Metals, Block clustering, PLS, Soils, Vegetables, Snails, Trophic chain

## Abstract

**Background:**

Given the serious threats posed to terrestrial ecosystems by industrial contamination, environmental monitoring is a standard procedure used for assessing the current status of an environment or trends in environmental parameters. Measurement of metal concentrations at different trophic levels followed by their statistical analysis using exploratory multivariate methods can provide meaningful information on the status of environmental quality. In this context, the present paper proposes a novel chemometric approach to standard statistical methods by combining the Block clustering with Partial least square (PLS) analysis to investigate the accumulation patterns of metals in anthropized terrestrial ecosystems. The present study focused on copper, zinc, manganese, iron, cobalt, cadmium, nickel, and lead transfer along a soil-plant-snai food chain, and the hepatopancreas of the Roman snail (*Helix pomatia*) was used as a biological end-point of metal accumulation.

**Results:**

Block clustering deliniates between the areas exposed to industrial and vehicular contamination. The toxic metals have similar distributions in the nettle leaves and snail hepatopancreas. PLS analysis showed that (1) zinc and copper concentrations at the lower trophic levels are the most important latent factors that contribute to metal accumulation in land snails; (2) cadmium and lead are the main determinants of pollution pattern in areas exposed to industrial contamination; (3) at the sites located near roads lead is the most threatfull metal for terrestrial ecosystems.

**Conclusion:**

There were three major benefits by applying block clustering with PLS for processing the obtained data: firstly, it helped in grouping sites depending on the type of contamination. Secondly, it was valuable for identifying the latent factors that contribute the most to metal accumulation in land snails. Finally, it optimized the number and type of data that are best for monitoring the status of metallic contamination in terrestrial ecosystems exposed to different kinds of anthropic polution.

## Background

In recent years it has become increasingly clear that industrial contamination is leading to serious threats to terrestrial ecosystems, thus endangering human and environmental health. Generally speaking, industrial contamination is related to any type of waste released into the environment from human industrial activities [1]. Metal contamination is, however, of particular interest because of metal highly toxic properties and their potential side-effects on ecosystem function and integrity [2]. Although this form of contamination dates back to antiquity, widespread industrial contamination accelerated rapidly with the start of the Industrial Revolution (the 1800s) and is currently regarded to be a serious problem in many countries [1]. Among the major sources of metal release we mentione several key human activities, such as mining operations, siderurgy, burning coal and oil in power plants, chemical industry, vehicular traffic, and intensive agriculture [1].

In this context, environmental monitoring is a standard procedure used for assessing the current status of an environment or trends in environmental parameters [2]. To determine the risks posed by metals on terrestrial ecosystems one should understand their fate along food chains. Briefly, metals are easily accumulated in soils, wherein they may persist over long periods of time [3]. The transfer process begins with the uptake of metals by the primary producers (green plants and bacteria), and continues to the next trophic level, the primary consumers (i.e., herbivores). Measurement of metal concentrations at these trophic levels can provide meaningful information concerning the status of environmental quality and ecosystem health at a specific moment of time [2,4], but only if the large amount of data resulted from such work are handled using appropriate chemometric approaches. Therefore, the present article deals with the most appropriate statistical methods to understand the factors contributing to contamination and metal accumulation in terrestrial ecosystems. Such questions are important in modern environmental science, especially for preliminary environmental impact assessment when researchers use descriptive applications to identify the underlying relationships between metal concentrations at different/same trophic levels.

To this end, many studies have relied on exploratory multivariate analysis to extract reliable information for environmental quality assessment [5-8]. In most cases the research question of interest in environmental chemistry and monitoring is expressed in terms of variables and cases (observations). A commonly used method to assess the similarity among different cases/variables is Hierarchical cluster analysis (HCA), also known as Tree clustering. This statistical technique reveals natural grouping (or clusters) within relatively large data sets based on measured characteristics. The graphical output is a dendrogram that shows how variables/cases are merged on one axis, whereas the other axis gives the distance at which any two clusters are joined [9]. However, this statistical technique does not allow environmental researchers to simultaneously merge the grouping of both cases and variables. The clustering of both by applying two-way joining clustering (syn. Block clustering) may yield relevant results not only for detecting clusters of cases with a similar magnitude of the measured variables, but also to explore the underlying relationships between these variables. Therefore, we propose that Block clustering (BC) may provide an interesting and powerful statistical approach in environmental monitoring if the researchers may want to simultaneously identify the similarity between different cases and variables.

When investigating large sets of data is beneficial to reduce their dimensionality in order to improve the efficiency and accuracy of data analysis [10]. Principal component analysis (PCA) is commonly used for this purpose in environmental research [7,11,12], but does not allow scientists to separate between the predictor and response variables [9]. Another statistical method, Exploratory factor analysis (EFA), uncovers the underlying structure for large sets of variables based on the shared variances among factors [13,14], but is sensitive to sample size, i.e., the sample size must be at least three-fold higher than the number of variables [15]. Partial least square (PLS) may represent a solution where such multivariate methods fail. This technique is routinely used in chemometric analysis when a large number of independent variables (>1000) are obtained with respect to a small number of samples (10 to 100) [16]. Depending on the objective of the study, PLS can serve either as a principal component technique, correlation technique, path modeling technique, or canonical correlation technique [17]. Overall, we suggest that this statistical method is a potential approach in environmental monitoring surveys for exploratory modeling of data sets with a large number of variables, but a moderate sample size (n = 20–50). Such situations are often encountered in baseline field surveys, which document the environmental conditions that exist at a specific moment in time to provide background in case of unknown changes in the future [18].

The present study enlarges our earlier survey [19] to include four additional metals (i.e., Mn, Fe, Ni, Co in addition to Cu, Zn, Cd, Pb) when investigating metal accumulation along soil-plant-snail food chain. These metals were chosen because they are known to serve as vital and/or toxic elements, depending on concentration, chemical and physical form. Nickel is regarded as having no obvious physiological role in plants and animals [20]. In contrast, manganese, iron, and cobalt act mainly as essential micronutrients, but their occurrence at high levels can represents a potentially serious hazard for environmental health [20]. This is of particular interest for Co and Mn, which, together with Ni, rank among the most dangerous 200 chemical compounds released in the environment from human activities according to the 2011 Substance Priority List of the US Agency for Toxic Substances and Disease Registry [21].

The Roman snail (*Helix pomatia*) was considered in the present study because this terrestrial gastropod concentrates high metal levels in its soft tissues without revealing any major metabolical disorders and serves as a major herbivore in terrestrial ecosystems [19]. The main purpose of this paper was to introduce a novel chemometric approach in environmental monitoring for understanding the similarities among sites/cases and determining the principal latent factors that influence metal accumulation in biological end-points by combining Block clustering with PLS and using a soil-plant snail food chain as study system.

## Results and disscusions

**Figure 1:**
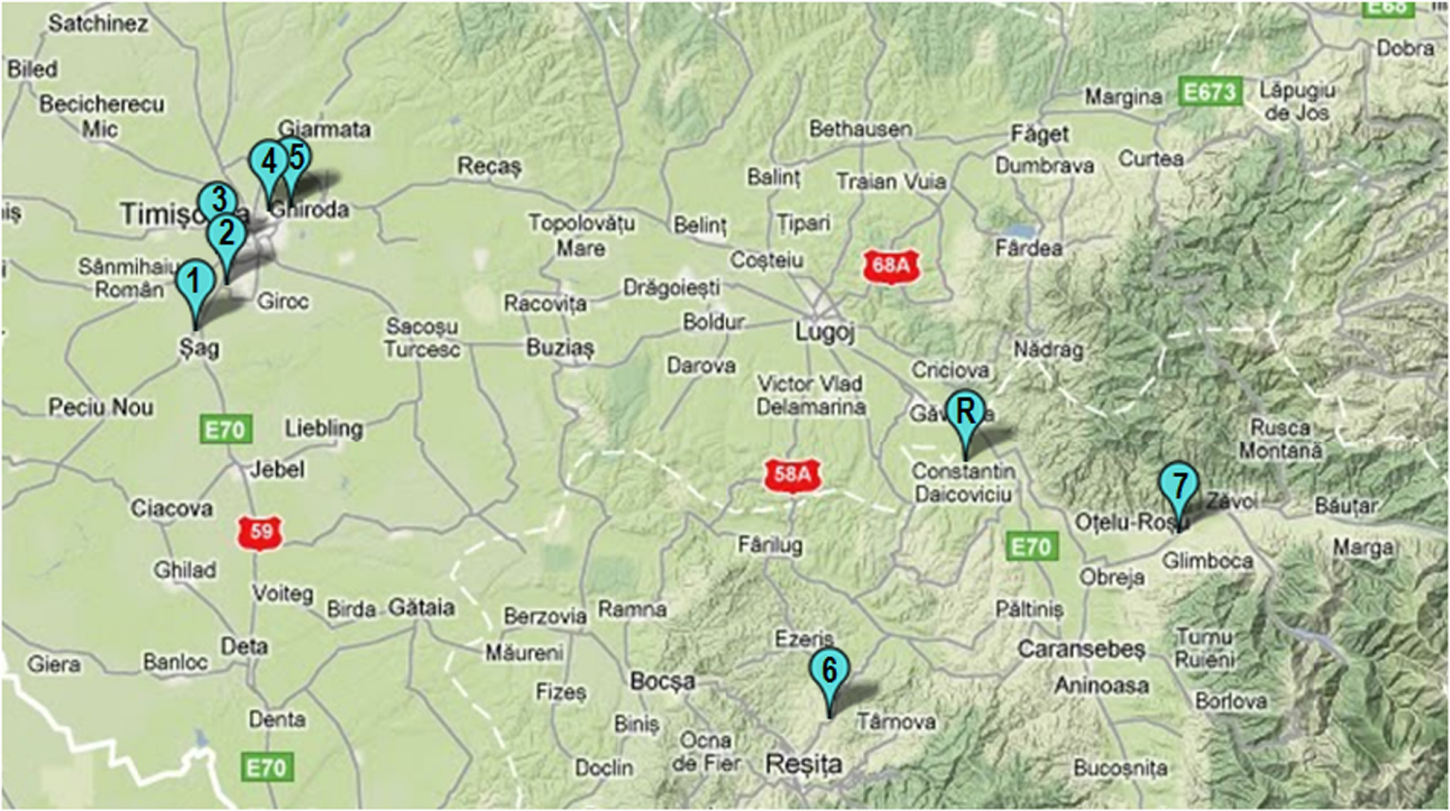
**General map showing the locations of soil, nettle leaves, and snail sampling sites. ***Legend*. 1–site THM1 (45.6765° lat. N; 21.1626° long. E); 2–site THM2 (45.7112° lat. N; 21.1968° long. E); 3–site THM3 (45.7420° lat. N; 21.1886° long. E); 4–site THM4 (45.7763° lat. N; 21.2517° long. E); 5–site THM5 (45.7787° lat. N; 21.2762° long. E); 6–site THM6 (45.3496° lat. N; 21.9196° long. E); 7–site THM7 (45.5053 lat. N; 22.3371 long. E); R–site THR (45.5667° lat. N; 22.0812° long. E).

**Figure 2:**
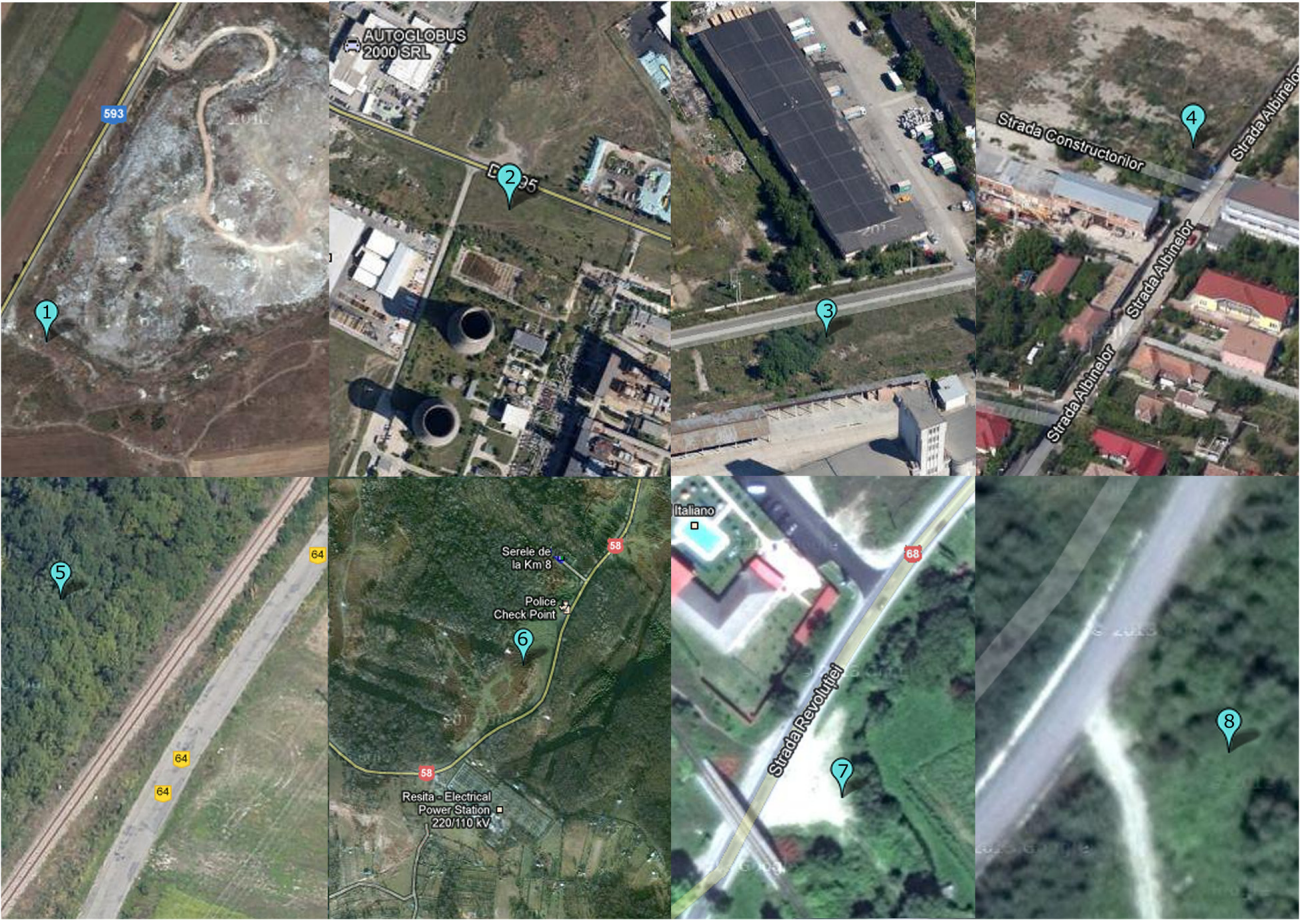
**Detailed map showing the locations of soil, nettle leaves, and snail sampling sites (source: Google Maps). ***Legend.* 1–site THM1 (45.6765° lat. N; 21.1626° long. E); 2–site THM2 (45.7112° lat. N; 21.1968° long. E); 3–site THM3 (45.7420° lat. N; 21.1886° long. E); 4–site THM4 (45.7763° lat. N; 21.2517° long. E); 5–site THM5 (45.7787° lat. N; 21.2762° long. E); 6–site THM6 (45.3496° lat. N; 21.9196° long. E); 7–site THM7 (45.5053 lat. N; 22.3371 long. E); R–site THR (45.5667° lat. N ; 22.0812° long. E).

The levels to which metals accumulate at different trophic levels, the normal content (NC) and alert threshold level (ATV) in soil for each investigated metal are shown as absolute values in Table 1. The standardized values at each trophic levels were normally distributed for all investigated metals (p > 0.05). It was found that the concentrations of metals in soils were within the normal levels at all sites, excepting Cd which occasionally did exceed the reference value from the Romanian Soil Quality regulations [22], but did not reach the corresponding alert threeshold level (ATV). These results showed that anthropic activities have a relatively low impact on soil metal concentrations in the study areas (Figures 1 and 2).

**Table 1:** **Metal concentrations in soils, nettle leaves, and snail hepatopancreas in the study area (expressed as mg kg**^**-1 **^**d.w.)**

	**Cu**	**Zn**	**Mn**	**Fe**	**Cd**	**Co**	**Ni**	**Pb**
	***Snail hepatopancreas***							
THR	8.219 ± 0.473	68.528 ± 4.648	100.439 ± 9.319	93.765 ± 10.303	0.526 ± 0.129	0.465 ± 0.045	0.557 ± 0.193	0.501 ± 0.085
THM1	23.063 ± 1.552	169.787 ± 16.821	104.351 ± 21.501	153.263 ± 19.119	17.196 ± 1.672	0.980 ± 0.294	1.156 ± 0.153	2.652 ± 0.707
THM2	25.984 ± 2.551	110.353 ± 5.137	64.130 ± 18.150	164.782 ± 3.515	3.231 ± 0.518	0.649 ± 0.356	1.041 ± 0.041	1.701 ± 0.821
THM3	9.684 ± 2.278	51.319 ± 2.957	32.690 ± 9.779	56.587 ± 6.224	0.529 ± 0.075	0.470 ± 0.260	0.560 ± 0.117	0.673 ± 0.333
THM4	8.610 ± 1.839	80.455 ± 1.763	68.212 ± 2.234	72.244 ± 8.659	1.410 ± 0.261	0.528 ± 0.161	0.552 ± 0.115	0.555 ± 0.125
THM5	33.071 ± 2.226	80.683 ± 7.994	55.347 ± 11.404	122.609 ± 15.295	3.675 ± 0.357	0.722 ± 0.216	1.180 ± 0.156	1.427 ± 0.380
THM6	24.005 ± 4.589	148.896 ± 13.544	131.322 ± 24.893	143.849 ± 19.170	4.792 ± 0.582	0.907 ± 0.199	1.085 ± 0.178	1.282 ± 0.093
THM7	9.632 ± 0.450	105.618 ± 9.972	84.592 ± 7.220	92.227 ± 10.907	5.914 ± 1.517	0.654 ± 0.048	0.728 ± 0.172	0.713 ± 0.192
MVL	17.783 ± 9.586	101.955 ± 39.471	80.135 ± 32.484	112.416 ± 39.343	4.659 ± 5.239	0.672 ± 0.261	0.858 ± 0.299	1.188 ± 0.794
	***Nettle leaves***							
THR	1.343 ± 0.039	6.782 ± 2.515	20.488 ± 4.036	22.439 ± 3.131	0.087 ± 0.009	0.143 ± 0.089	0.372 ± 0.256	0.298 ± 0.032
THM1	8.894 ± 1.735	21.713 ± 2.808	30.865 ± 1.429	13.657 ± 2.454	2.882 ± 0.447	0.206 ± 0.042	0.447 ± 0.147	11.934 ± 2.134
THM2	19.387 ± 3.863	34.957 ± 5.726	16.540 ± 3.613	22.524 ± 5.406	2.612 ± 0.789	0.214 ± 0.033	0.363 ± 0.242	9.779 ± 0.839
THM3	1.196 ± 0.342	32.563 ± 6.200	13.001 ± 2.716	7.547 ± 1.762	0.643 ± 0.203	0.189 ± 0.046	0.283 ± 0.117	24.545 ± 3.606
THM4	3.458 ± 0.412	33.900 ± 6.070	26.048 ± 3.524	9.136 ± 1.111	2.769 ± 0.875	0.203 ± 0.042	0.359 ± 0.152	9.470 ± 1.926
THM5	3.130 ± 0.611	8.191 ± 1.059	4.093 ± 0.189	23.666 ± 4.253	0.074 ± 0.011	0.154 ± 0.032	0.333 ± 0.109	0.240 ± 0.043
THM6	2.485 ± 0.191	9.686 ± 2.302	14.454 ± 0.652	17.359 ± 1.558	0.066 ± 0.025	0.151 ± 0.037	0.335 ± 0.206	2.368 ± 0.508
THM7	1.959 ± 0.329	13.093 ± 1.401	8.191 ± 0.423	18.629 ± 3.403	0.086 ± 0.018	0.154 ± 0.077	0.435 ± 0.068	0.203 ± 0.062
MVL	5.232 ± 6.081	20.111 ± 12.156	16.710 ± 8.750	16.870 ± 6.499	1.152 ±1.337	0.178 ± 0.052	0.366 ± 0.154	7.355 ± 8.221
	***Soil***							
THR	2.516 ± 1.343	9.453 ± 1.176	48.188 ± 8.752	115.309 ± 12.563	0.151 ± 0.019	1.421 ± 0.202	1.027 ± 0.178	4,758 ± 0,436
THM1	14.364 ± 2.881	32.481 ± 3.891	54.112 ± 9.427	163.388 ± 18.880	2.048 ± 0.369	2.198 ± 0.633	4.253 ± 0.393	14,766 ± 2,673
THM2	26.832 ± 3.309	52.293 ± 5.911	51.890 ± 4.267	199.860 ± 24.852	1.088 ± 0.176	2.706 ± 0.449	5.507 ± 0.493	39,336 ± 1,040
THM3	11.950 ± 2.359	48.711 ± 3.496	50.323 ± 4.773	168.028 ± 8.018	0.713 ± 0.257	2.001 ± 0.678	3.420 ± 0.243	21,414 ± 1,592
THM4	15.196 ± 2.885	50.713 ± 11.301	58.380 ± 10.557	16.519 ± 1.824	0.643 ± 0.092	2.395 ± 0.511	4.592 ± 1.220	52,954 ± 7,241
THM5	8.714 ± 1.748	22.588 ± 2.706	51.598 ± 8.989	162.102 ± 18.731	2.769 ± 0.499	1.784 ± 0.712	3.329 ± 0.308	10,409 ± 1,884
THM6	3.074 ± 0.468	13.472 ± 1.187	47.388 ± 3.667	111.410 ± 11.260	2.124 ± 0.183	1.858 ± 0.619	3.621 ± 0.976	11,589 ± 0,818
THM7	8.546 ± 1.865	29.051 ± 1.187	58.507 ± 9.479	179.529 ± 9.946	0.413 ± 0.066	1.639 ± 0.473	2.438 ± 0.366	10,604 ± 1,938
MVL	11.399 ± 7.687	32.345 ± 2.553	52.548 ± 7.734	139.518 ± 56.902	1.244 ± 0.932	2.000 ± 0.613	3.523 ± 1.407	20,729 ± 16,215
NC	20	100	900	-	1	15	20	20
ATV	100	300	1500	-	3	50	75	50

*Abbreviations:* THM1 - Timisoara city Landfill (Parta-Sag/Timis county); THM2 - South Industrial Platform (Timisoara/Timis county); THM3 - North Industrial Platform (Timisoara/Timis county); THM4 - East Industrial Platform (Timisoara/Timis county); THM5 - Timisoara, Communal Road 64 (DC64) (Timisoara/Timis county).; THM6 - National Road Resita-Caransebes (DN58) (Caras-Severin county); THM7 - Principal Street (Otelu Rosu/Caras-Severin county); THR - Salbagelu Nou (Caras-Severin county); NC–normal metal content in the soil; ATV–alert threshold level for metal concentrations in the soil; MVL–average metal level for all sites taken together.

**Figure 3:**
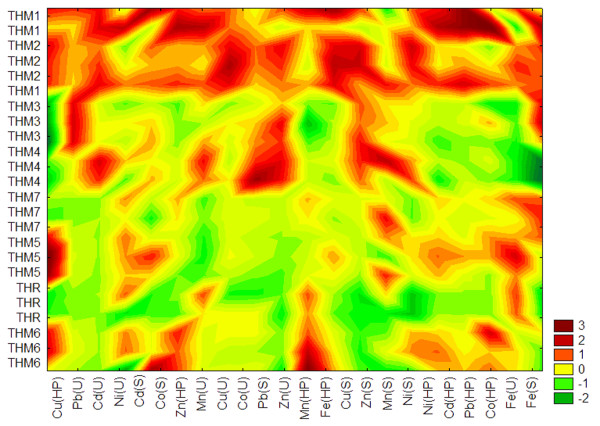
**Block clustering of the metal concentrations in soil, nettle leaves, and snail hepatopancreas. ***Legend.* Cu(S)–copper concentrations in soil; Cu(U)–copper concentrations in nettle leaves; Cu(HP)–copper concentrations in snail hepatopancreas; Zn(S)–zinc concentrations in soil; Zn(U)–zinc concentrations in nettle leaves; Zn(HP)–zinc concentrations in snail hepatopancreas; Mn(S)–manganese concentrations in soil; Mn (U)–manganese concentrations in nettle leaves; Mn(HP)–manganese concentrations in snail hepatopancreas; Fe(S)–iron concentrations in soil; Fe (U)–iron concentrations in nettle leaves; Fe (HP)–iron concentrations in snail hepatopancreas; Cd(S)–cadmium concentrations in soil; Cd(U)–cadmium concentrations in nettle leaves; Cd(HP)–cadmium concentrations in snail hepatopancreas; Co(S)–cobalt concentrations in soil; Co(U)–cobalt concentrations in nettle leaves; Co(HP)–cobalt concentrations in snail hepatopancreas; Co(S)–nickel concentrations in soil; Co(U)–nickel concentrations in nettle leaves; Co(HP)–nickel concentrations in snail hepatopancreas; Pb(S)–lead concentrations in soil; Pb(U)–lead concentrations in nettle leaves; Pb(HP)–lead concentrations in snail hepatopancreas.

The dendrogram groups the environmental variables on the x-axis using the squared Euclidean distance as a criterion of similarity (Figure 3). At the abiotic level we can observe similar distributions in the soil among the total concentrations for Cu, Zn, Mn, and Ni, and Cd and Co, respectively. Such relationships may reflect the fact that these metals share common anthropogenic sources, such as combustion of coal and heavy fuel oil, municipal waste incineration, vehicular traffic, chemical plants, ferrous and non-ferrous metal production [23]. As a result of having no functional role in plants and terrestrial gastropods [24,25], the measured values for Ni, Cd, and Pb fall close to each other on the x-axis both in the nettle leaves and snail hepatopancreas. Similar associations are also found between the essential trace metals. Because Cu, Co, and Mn are essential regulators of plant growth and development [24], they are clustered near each other in the nettle leaves. There is a close association between the Mn and Fe accumulation in the hepatopancreas; this is mainly related to the fact that these essential microelements follow similar metabolic pathways in land snails [25]. Copper is exclusively regulated in *H. pomatia* by a specific metallothionein [26], and therefore, its distribution in the hepatopancreas is independent of any other metals. This element is essential for land snails because it is a a component of the chromoprotein hemocyanin, which is essential to their respiration [25].

The y-axis clusters the sites with similar distribution of metals at different trophic levels (Figure 3). Combining the x- and y-axes reveals information about the underlying similarities among sites, which cannot be provided by applying the Tree Clustering method. Green colors represent lower than average values and yellow to brown the opposite. The first sampling point (THM1) is located near the Sag-Parta landfill, whereas the second sampling point lies within the South Industrial Platform, about 100 m far away from the Timisoara Sud Power Plant. As a result of being located close one to each other (about 1km), these sites showed a similar pattern of metal accumulation at all trophic levels, which is associated with exposure to the same source of environmental pollution (i.e., South Industrial Platform Timisoara).

The site THM1 regularly exhibited the highest metal concentrations among different locations, irrespective of trophic level. This site does not have engineered systems for collecting landfill leachate or gases, and as a consequence, it is considered as a class B landfill that is suitable to accept only general domestic and commercial waste [27]. Although this landfill was officially closed in 2009 [28], it remains a serious pollution hotspot in the Timisoara area, as shown by our results (Table 1). Similarly, the site THM2 revealed higher metal concentrations along soil-plant-snail food chain as compared to the other investigated sites. This site lies near multiple sources of anthropic pollution, and as a consequence, such findings are not surprising. Our results are in line with recent studies, which found high levels of metals in vegetables from areas adjacent to the South Industrial Platform Timisoara [29].

The third sampling site (THM3) lies near the former “Solventul Timisoara” petrochemical works. This site fall close to the fourth sampling point (THM 4), which is located within the East Industrial Platform Timisoara. These locations share a similar pattern of Zn accumulation in the soil and nettle leaves (Figure 3). Because both sites are known for long-term exposure to chemical and petrochemical industries, the routine use of Zn compounds in these industries (e.g., zinc oxide as pigment in paint industry or catalysts in the manufacture of rubber) may therefore explain our findings [30].

The fourth sampling point (THM5) is placed in a wooden area, near the Communal Road DC64 (Timisoara-Ghiroda). The seventh site (THM7) lies in the city of Otelu Rosu, about 50 m far away from the National Road DN68 (Caransebes–Hateg), and 150 m of the former Otelu Rosu steel works, respectively. Interestingly, these two sites are clustered near each other on the vertical axis although they are placed in different counties (i.e., the site THM5 lies more than 100 km away from the site THM7). Although manganese is one of the most abundant metals in soils, its deposition in soil was also shown to be associated with trafficked roads [31]; therefore, the moderate concentrations of Mn that are found in the soil at both sites may be linked to a similar intensity of vehicular traffic along the DC64 and DN68 roads (Figure 3).

The reference site (THR) corresponds to an area located away from major sources of pollution [32], about 100 m away from the communal road which connects the National Road DN58 to the village of Salbagelu Nou, whereas the sixth site (THM6) lies along the National Road DN58 (Resita-Caransebes). The latter location generally shows higher metal levels than the site THR, which are related to the cumulative action of long-term exposure to vehicular traffic and metallic contamination (Resita steel works). Overall, we can observe that the dendrogram obtained by using the Block clustering method separates the sites in two groups (Figure 3). The first group (G1) contains the sites located on industrial platforms from Timisoara (i.e., THM1-THM4), whereas the second group (G2) includes the sites located within 100 m away from roads with different intensity of vehicular traffic (i.e., THM5-THM7, THR).

**Figure 4:**
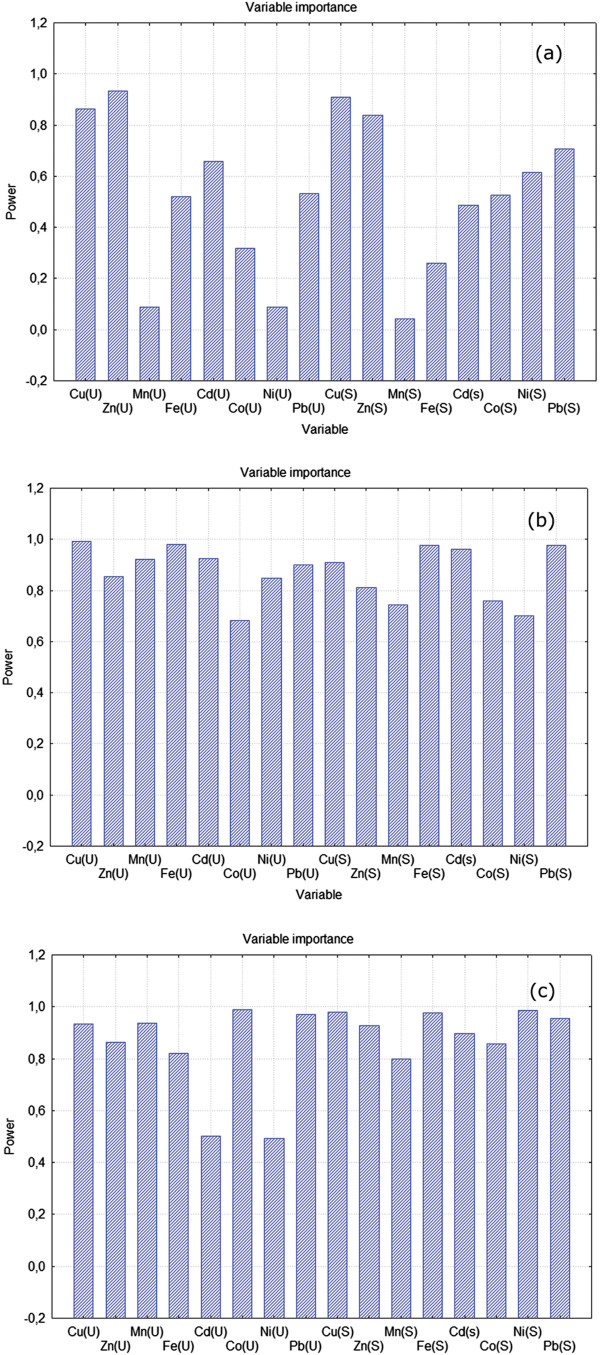
VIP values for the exploratory PLS model for all sites (a), the G1 sites(b) and the G2 sites (c).

The exploratory PLS analysis for all sites extracted two significant latent factors (Table 2a), which explained 58.11% of the variance of response variables and 52.41% of the variance of predictor variables, respectively. The weights of predictor variables determine VIP (Variable Importance in the Projection), which shows the statistical contribution of the variable in fitting the PLS model. Variables for which the VIP scores are less than 0.8 are regarded to be small [33], and therefore, one can conclude that copper and zinc concentrations in soil and nettle leaves are contributing the most to trace metal accumulation pattern in land snails (Figure 4a). These findings are consistent with land snail physiology, wherein copper is a key player in metabolic activities [34].

**Table 2:** Results of the exploratory PLS analysis for all sites (a), the G1 sites(b) and the G2 sites (c)

**Factor**	**R**^**2**^**X**	**R**^**2**^**X(Cumul.)**	**Eigenvalues**	**R**^**2**^**Y**	**R**^**2**^**Y(Cumul.)**
**(a)**					
1	0.169	0.169	2.201	0.473	0.473
2	0.355	0.524	4.671	0.108	0.581
**(b)**					
1	0.289	0.289	4.436	0.507	0.507
2	0.254	0.542	3.773	0.173	0.680
3	0.130	0.672	1.897	0.182	0.862
4	0.131	0.803	1.952	0.040	0.902
5	0.065	0.868	1.015	0.058	0.960
**(c)**					
1	0.269	0.269	4.105	0.693	0.693
2	0.256	0.525	3.976	0.100	0.793
3	0.110	0.635	1.694	0.095	0.889
4	0.182	0.817	2.853	0.039	0.928
5	0.054	0.871	0.638	0.022	0.951

*Abbreviations:* R^2^X–percentage of X variance explained by each PLS factor; R^2^X(Cumul.)–cumulative modeled variation in X; R^2^Y - percentage of Y variance explained by each PLS factor; R^2^Y(Cumul.) - cumulative modeled variation in Y.

We have reran the PLS analysis with all predictor variables on each of the two groups that were obtained by applying Block clustering. The physiological metals (i.e., Cu, Zn, Mn, Fe, Co) were removed from our analysis because the levels to which these microelements accumulate in soils were generally within NC values at all sites (Table 1). Among the toxic metals (e.g., Ni, Cd, Pb), priority was given to those elements which showed high VIP scores (VIP > 0.8) for concentrations in both the soil and nettle leaves. Reducing the number of independent variables implies that fewer terms are needed in the expansion to find the metals with the highest/lowest risk on environmental and human health in the study areas.

The PLS model for the G1 sites had five significant factors, which explained 87.12% variance of response variables and 95.05% of the variance of predictor variables, respectively (Table 2b). The importance of most predictor variables was high (VIP > 0.8), excepting Mn and Ni levels in the soil, and Co content in the soil and *U.dioica* leaves (Figure 4b). It can be seen from the Figure 4b that Cd and Pb concentrations, in contrast with Ni levels, have high VIP scores in both the soil and nettle leaves. Therefore, the G1 sites should be closely monitored in the future with respect to Cd and Pb accumulation along terrestrial food chains. Our results are consistent with recent studies, which found that Cd and Pb are the main determinants of pollution pattern in the Timisoara area [29,35].

For the G2 sites, the PLS analysis extracted five significant factors, which accounted for 86.76% of the variation of response variables and 96.04% of the variation of predictor variables, respectively (Table 2c). All physiological metals showed high VIP scores in the soil and nettle leaves (Figure 4c). Among toxic metals, Pb was the only element for which the concentrations in soils and nettle leaves displayed high VIP scores (Figure 4c). These findings are not surprising since Pb is among the most common heavy metals (together with Cu and Zn) released from vehicular traffic [36].

## Conclusions

The legacy of metals released to the environment from human activities puts increasing pressure on terrestrial ecosystems from anthropized areas. To this end, not only finding novel analytical methods for determining the degree of environmental contamination, but also employing new statistical approaches for analyzing environmental data provide scientists with powerful tools in environmental monitoring and assessment.

In the present study, we show that applying Block clustering with PLS analysis is simple and intuitive procedure that allows researchers to:

– overcome the drawbacks imposed by the graphical representation of environmental variables. Although such charts are useful in environmental monitoring, they hide what the data tell us when too many variables are illustrated within the same chart;

– group sites with similar patterns of metal accumulation at different trophic levels, thus allowing environmental researchers to separate the study areas depending on the type of contamination and to understand the underlying similarities among them;

– select the most significant latent factors (based on VIP values) which explain metal accumulation in biological end-points;

– optimize the analytical procedures by selecting for future investigations only the toxic metals with high VIP scores, thus reducing the analytical costs; in this case, the emphasis of contamination with Cd and Pb on industrial platforms near Timisoara and with Pb near roads;

– provide a benchmark for building exploratory models with potential applications in environmental monitoring surveys when researchers have to deal with data sets having a large number of variables, but a small sample size.

## Experimental

Detailed description concerning the location of sampling sites, the preparation of samples, and the analysis of metals are provided in our previous work [19]. Briefly, the samples were collected in triplicate for each trophic level (soil, nettle, snail) from eight sites located in the western part of Romania, in the Banat area (Timis and Caras-Severin counties). All locations have been exposed to long-term industrial pollution (> 30 years), and lie at most 10 km away from former and/or actual sources of anthropic contamination. The reference area (site THR), the vilage of Salbagelu Nou (Caras-Severin county), is located in a non-polluted area, with less industry [31]. The sites THM1-THM5 are located around the city of Timisoara, the most populated and industrialized city from the Banat area. The sites THM6 and THM7 are located along trafficked roads, in an area which was exposed for more than two centuries to the impact of metallurgical industry [37]. For each sampling plot at least 60 newly matured *Helix pomatia* specimens were collected, fasted for 48 h and sacrificed by freezing (at −20°C). To provide homogeneous samples the snails were calibrated based on the shell height, which was shown to serve as a more accurate predictor of snail size as compared to the shell width [19]. The measurements were performed with a digital caliper to the nearest 0.01 mm. After defrosting, the whole soft body was removed from the shell and the viscera and the foot were separated. Only the snail hepatopancreas was considered in the present study because this organ was found to serve as the main end-point of metal accumulation for *H. pomatia*[19]. The samples were analysed in triplicate for each location, and 20 snails were used for each batch.

The selected food chain included nettle as the main food source of Roman snail (*Helix pomatia*) based on observations of snail feeding habits in investigated areas. For each location three samples from the top leaves were collected, rinsed in distilled water to wash off potential air pollutants, and then oven dried at 105°C to constant weight. The samples were crushed with a mortar, passed through a 2 mm sieve, and preserved in self-sealing sterile paper pouches at room temperature (t = 22°C). The soil samples were collected (25 g/sample in triplicate) from the top 15 cm layer after removal of vegetation (grass). After removing roots and litter, they were dried (t = 22°C, 7 days), disaggregated, homogenized before being sieved to 2 mm (soil metal concentration analysis), and then stored at ambient temperature (t = 22°C) for further analysis [19].

The extraction of metals was performed by wet extraction (in HNO_3_ 0.5N) for the soil samples, and by ash digestion (with HNO_3_ 0.5N) for the nettle and snail samples. The ash was obtained by burning the nettle and snail samples for 8 h, at 550°C, in the muffle furnace (Nabertherm B150, Lilienthal, Germany). The metals were determined by flame atomic absorption spectrometry (FAAS) with high resolution continuum source (Model ContrAA 300, Analytik Jena, Germany), in the Environmental Research Test Laboratory, Banat’s University of Agricultural Sciences and Veterinary Medicine from Timisoara, Romania. This spectroscopic method was chosen because it is a fast and easy technique with an extremely high sensitivity for elements like Pb, Cd, Cu and Cr. NCS Certified Reference Material-DC 85104a and 85105a (China National Analysis Center for Iron&Steel), were analyzed for quality assurance. Percent recovery means were: Fe (92%), Mn (95%), Zn (102%), Cu (105%), Ni (99%), Pb (94%), Cd (105%), Co (98%). The variation coefficients were below 10%. Detection limits (μg/g) were determined by the calibration curve method: Fe (0.15), Mn (0.19), Zn (0.43), Cu (0.13), Ni (0.14), Cd (0.01), Pb (0.05), Co (0.07).

### Statistical analysis

Because the metal concentrations differed by several degrees of magnitude, the measured values were standardized as follows:

$$\mathrm{STV}=\left(\mathrm{RV}-\mathrm{MV}\right)/\mathrm{STD}$$

where STV defines the standardized value, RV the raw value, MV the mean value, and STD the standard deviation. As a result, the data were displayed on scale from −1 to +1.

The subsequent data were then checked for normality using both Anderson-Darling test (for comparing distribution functions) and Jaques-Bera test (for comparing between kurtosis and skewness of a function). We performed a cluster analysis (Block clustering) to identify the sites with similar patterns of metal accumulation at different trophic levels and to explore relationships between these variables. Exploratory PLS analysis was used for assessing the principal latent variables (factors) underlying metal accumulation in the snail hepatopanceas. The analysis was carried out for all sites taken together, as well as separately for each cluster of sites obtained by applying the Block clustering method. Metal concentrations in the soil and nettle leaves were considered as independent/predictor variables (X of the PLS matrix), whereas their levels in the snail hepatopancreas were taken into account as dependent/outcome variables (Y of the PLS matrix). To validate the PLS model, the number of extracted factors was chosen through 10-fold cross validation, i.e. fitting the model to part of the data and minimizing the prediction error for the unfitted part [17]. Statistical analyses were performed by using the Statistica 10 software package [38]. All data are presented as the mean ± SD for the absolute measured values.

## Abbreviations

PLS: Partial least square analysis; VIP: Variable importance in the projection; THR: Reference sampling site; THM1-THM7: Sampling sites 1-7; NC: Normal content; ATV: The alert threshold level.

## Competing interests

The authors declare that they have no competing interests.

## Authors’ contributions

DVN, DMB, IP, EP, SA, and IG have contributed mainly to the study design, collection of data, sampling of soil, vegetation, and snails, chemical analyses, interpretation of results and preparation of paper. All authors read and approved the final manuscript.
